# Prospective longitudinal course of cognition in older subjects with mild parkinsonian signs

**DOI:** 10.1186/s13195-016-0209-7

**Published:** 2016-10-10

**Authors:** Stefanie Lerche, Kathrin Brockmann, Andrea Pilotto, Isabel Wurster, Ulrike Sünkel, Markus A. Hobert, Anna-Katharina von Thaler, Claudia Schulte, Erik Stoops, Hugo Vanderstichele, Victor Herbst, Britta Brix, Gerhard W. Eschweiler, Florian G. Metzger, Walter Maetzler, Daniela Berg

**Affiliations:** 1 Center of Neurology, Department of Neurodegeneration and Hertie-Institute for Clinical Brain Research, University of Tuebingen, Hoppe Seyler-Strasse 3, Tuebingen, 72076 Germany; 2 German Center for Neurodegenerative Diseases, University of Tuebingen, Otfried-Müller-Straße 27, Tuebingen, 72076 Germany; 3 Neurology Unit, Department of Clinical and Experimental Sciences, University of Brescia, Brescia, Italy; 4 ADx NeuroSciences, Gent, 9052 Belgium; 5 EUROIMMUN Medizinische Labordiagnostika AG, Lübeck, Germany; 6 Geriatric Center at the University Hospital of Tübingen, Tübingen, Germany; 7 Department of Psychiatry and Psychotherapy, University Hospital of Tübingen, Tübingen, Germany

**Keywords:** Amyloid-beta, Dementia, Prospective, Longitudinal, Cohort study

## Abstract

**Background:**

Mild parkinsonian signs (MPS) are common in older people and are associated with an increased risk of different neurodegenerative diseases. This study prospectively evaluates the longitudinal course of cognitive performance in older individuals with MPS.

**Methods:**

From the TREND study, 480 individuals neurologically healthy at baseline, aged between 50 and 80 years, with complete follow-up data for three assessments within a mean of 43.8 months, were included in this analysis. Participants underwent a detailed cognitive test battery, evaluation of prodromal markers for neurodegenerative diseases and history of vascular diseases at each study visit. In addition, plasma levels of amyloid-beta (Aβ)_1–40_ and Aβ_1–42_ were evaluated longitudinally.

**Results:**

In 52 (11 %) of the 480 participants, MPS could be detected at baseline. These individuals had cognitive deficits significantly more often compared with controls at each time point and their cognitive performance showed a steeper decline during follow-up. In addition, their levels of plasma Aβ_1–42_ were significantly lower than those of controls, and declined more rapidly over time.

**Conclusions:**

This longitudinal study shows that MPS are associated with cognitive decline and decrease in plasma Aβ_1–42_, possibly indicating an ongoing neurodegenerative process.

**Electronic supplementary material:**

The online version of this article (doi:10.1186/s13195-016-0209-7) contains supplementary material, which is available to authorized users.

## Background

Symptoms of bradykinesia, rigidity and tremor occurring in isolation as well as in combination are common in older people who do not meet the criteria for Parkinson’s disease (PD) or other neurodegenerative diseases [1, 2]. These are referred to as mild parkinsonian signs (MPS). Obviously these symptoms can be caused by a variety of non-neurological conditions as well as by neurological disorders. MPS have been shown to be associated with several different diseases and adverse health outcomes, such as PD, dementia, functional disability and mortality [2–5]. Three prospective studies have so far demonstrated an association between the presence of MPS in non-demented older adults and the development of incident dementia (including Alzheimer’s disease (AD)) during follow-up. Wilson et al. [6] showed already that a slight progression of MPS doubles the risk for developing AD. Further, Louis et al. [4] showed in a study with 1028 individuals that those with MPS at baseline had an increased risk of 56 % for developing incident dementia after a mean follow up of 5.6 years. In a large prospective study including 1851 individuals, the same group found dementia in 16 % of the MPS participants after a mean follow up of 3.7 years, with AD being the most common type (86 %) [7]. Further, there are several cross-sectional analyses showing an association between MPS and mild cognitive impairment in the “prodromal phase” of AD [8–10]. Taken together, there is evidence that MPS might be a “prodromal marker” for future incident dementia. The “prodromal phase” of a neurodegenerative disease is defined as the phase in which neurodegeneration is ongoing but a clinical diagnosis cannot be made. Symptoms of the prodromal phase comprise motor and non-motor markers (“marker” here refers to any disease indicator, whether a symptom, sign or biomarker).

An opportunity to better estimate the possible role of MPS as a prodromal stage of AD is the longitudinal evaluation of clinical and biofluid markers in individuals at risk for this form of dementia.

To the best of our knowledge, no study has yet evaluated individuals with MPS with regard to cognitive symptoms and biological/biochemical markers longitudinally with biennial evaluations. To address this issue we investigated a large cohort of healthy older individuals with and without MPS, longitudinally over a study period of 3.5 years, using a cognitive test battery, self-administered questionnaires for prodromal AD markers and co-morbidities and ultrasound for the measurement of the intima-media thickness (IMT) of the carotids. Moreover we analysed biomaterial of the participants for amyloid-beta (Aβ)_1–40_ and Aβ_1–42_ and apolipoprotein (*ApoE*) genotypes.

## Methods

### Study population

The TREND study is a prospective follow-up study initiated in 2009, which included individuals aged ≥50 years at baseline and provides biennial assessments until death/autopsy. For a detailed outline of the TREND study, as well as inclusion and exclusion criteria, see Gaenslen et al. [11]. A large assessment battery with mainly quantitative, unobtrusive measurements designed to be repeated easily and objectively is being applied. To make sure that there is no bias in data acquisition, all investigators are blinded to the results of all other examinations. The TREND study initially comprised 715 participants of whom 593 individuals had three evaluations, the prerequisite for being eligible for analyses in this study. Of these 593 individuals, those with complete datasets of neurological examination, a cognitive test battery and plasma levels of Aβ_1–40_ and Aβ_1–42_ were included in this analysis. In total, 480 individuals (52 with persistent MPS and 428 without) were analysed.

### Neurological examination

Each participant underwent a standardized neurological examination and the motor part of the revised Unified Parkinson’s Disease Rating Scale (MDS-UPDRS) applied by an experienced movement disorder specialist [12]. In accordance with other large-scale studies [13, 14], MPS were defined as present when any of the following conditions was met: at least two MDS-UPDRS III items ≥1; or at least one MDS-UPDRS III item ≥2; iii) at least one MDS-UPDRS rest tremor item ≥1. Only individuals in whom MPS were present at all three time points were defined as individuals with persistent MPS. Individuals in whom the results of the neurological examination revealed PD according to the UK Brain Bank Criteria were excluded from this analysis [15].

### Assessment of demographics and medical history

Each participant underwent a structured medical interview including demographics, medical history and medication. A family history questionnaire was used to obtain information on all first-degree and second-degree relatives of the participants. Vascular risk factors were assessed with a self-assembled medical questionnaire which includes questions about history of ischaemic stroke or transient ischaemic attack, heart attack, arrhythmia, angina pectoris, occlusive disease of the peripheral arteries, congestive heart failure, hypertension, hypercholesterolaemia and diabetes mellitus. This questionnaire allows one to rate the existence or absence of the afore-mentioned disturbances (“yes/no”). In addition, the intake of medication was recorded and used to verify the answers.

### Assessment of prodromal markers

#### Impaired olfaction

Olfaction was tested using the 16 Sniffin’ sticks battery (Burghart Medizintechnik, Germany) as described by Hummel et al. [16]. According to the suggestion of Hummel and colleagues, individuals identifying less than 75 % of odours correctly were classified as having hyposmia. Some of the 480 participants had co-morbidities prohibiting the correct determination of olfaction (report of allergic rhinitis/cold or nose surgery). At baseline, olfaction could not be tested in 24 participants (21 healthy controls and three individuals with persistent MPS).

#### Rapid eye movement sleep behaviour disorder

Presence of rapid eye movement sleep behaviour disorder (RBD) was determined by a self-administered RBD screening questionnaire (RBDSQ). The RBDSQ is a recently developed questionnaire, comprising 10 items to describe the most prominent clinical features of RBD [17]. Presence of RBD was accepted in participants with ≥5 points in the questionnaire (sensitivity 96 % and specificity 92 %).

#### Depression

Depressive symptoms were assessed with the Beck Depression Inventory I (BDI-I). The BDI-I is a 21-item self-report questionnaire, ranging from 0 to 63 (no depression, 0–10; mild to moderate depression, 11–17; severe depression, 18–63) [18].

### Intima-media thickness

Measurement of the right common carotid artery (CCA) was performed in the first follow-up using a 5–10 MHz linear array transducer (VF10-5; Siemens, Erlangen, Germany). Participants were examined in a supine position with their head tilted backwards. The CCA was differentiated from the internal jugular vein in a transverse plane, displayed in a longitudinal scan while the course of the CCA was followed up to the carotid bulb. IMT was measured 1 cm proximal of the carotid bulb using a fourfold magnification of the ultrasound image [19]. The IMT of the CCA was defined as the distance between the intima line and the media–adventitia border at the far wall of the CCA [20]. Measurements were documented and images were stored. IMT was defined as pathologic if the measurement was >1.0 mm [21, 22].

### Cognition

Cognition was tested using an established and standardized cognitive test battery. We used the German version of the extended Consortium to Establish a Registry for Alzheimer’s Disease neuropsychological battery (CERAD-Plus) [23]. The battery contains the following subtests: verbal fluency, Boston Naming Test, Mini Mental State Examination, word list memory, word list recall, word list recognition, constructional praxis, recall of constructional praxis, phonematic fluency and the Trail-Making Test Parts A and B (TMT-A and TMT-B). The TMT consists of two parts and evaluates executive function, cognitive flexibility and working memory [24, 25]. In TMT-A, subjects have to connect randomly spread numbers from 1 to 25 in ascending order. In TMT-B, participants are asked to connect randomly spread numbers (1–13) and letters (A–L) in alternating numeric and alphabetical order (1–A–2–B–3–C–…–13–L). In the case of an error, the examiner draws the attention of the participant to the error, to allow completion of the task without errors at the expense of additional time. The maximum time allowed is 180 s for TMT-A and 300 s for TMT-B. After this time the investigator discontinues the experiment [26]. TMT performance was calculated taking the time needed to perform TMT-B minus the time needed for TMT-A. This ΔTMT value prevents possible bias due to differences in upper extremity motor speed, simple sequencing, visual scanning and psychomotor functioning [24, 25]. The subtests of the CERAD-Plus battery were grouped into four domains: executive, memory, language and visuospatial. For the analysis of these domains, demographically adjusted *z* scores were used. The total score of the CERAD battery, ranging from 0 to 100, has been shown to provide an effective global measure of cognitive functioning [27].

### Biomaterial and analyses of biomarkers in plasma

Standard operating procedures were defined for the collection, preparation, storage and analysis of biomaterial obtained from the study participants.

#### Plasma Aβ_1–38_, Aβ_1−40_ and Aβ_1–42_ levels

EDTA plasma was centrifuged at 2000 × *g*, 4 °C for 10 min and stored at −80 °C within 60 min after collection. Plasma levels of Aβ_1–42_ and Aβ_1–40_ were quantified by EUROIMMUN Beta-Amyloid 1–42 Plasma ELISA and EUROIMMUN Beta-Amyloid 1–40 Plasma ELISA, respectively. Instead of a manual procedure, the automated workup on the ANALYZER-I (EUROIMMUN) was applied. Test specifications and characteristics for the methodology are shown in Additional file 1. For quality control (QC) purposes, QC samples were produced by pooling of EDTA plasma samples. Some samples were used native and others were spiked to higher concentrations with calibrator material. After aliquoting, samples were freeze dried for long-term stability. The samples were coded R2, R3, R6 and R8 and used over the three plasma amyloid assays. For test run monitoring, three QC samples were included (single testing) in each test run in parallel with the test samples. Only runs with available data points for the three samples were considered. The coloured blocks show the 95 % confidence interval (±2 SD). For details, see Additional file 2.

#### *ApoE* genotypes

Genomic DNA was extracted from EDTA blood using standardized protocols. *ApoE* genotypes were analysed by a multiplex SNaPshot assay (Applied Biosystems Life Technologies GmbH, Darmstadt, Germany) (PMID:15505371). All SNPs investigated were in Hardy–Weinberg equilibrium (data not shown).

### Statistical analysis

Statistical analysis was performed using SPSS 22.0 software for Windows (SPSS Inc., Chicago, IL, USA). Because of the asymmetric distribution between the two groups, differences of non-categorical data were evaluated with the Wilcoxon rank-sum test. The Fisher’s exact test was used for categorical data. Descriptive statistics are given either as median (range) and mean ± standard deviation for non-categorical data or as percentages of total for categorical data. CERAD total score, TMT and plasma levels of Aβ_1–40_ and Aβ_1–42_ have been evaluated for each time point adjusting for age, gender and educational levels (only cognition). For the CERAD subdomains, demographically adjusted *z* scores were used. Differences were considered significant at *p* < 0.05.

## Results

The 480 TREND participants had a mean duration of total follow-up of 43.8 ± 4.2 months (baseline (BL) to first follow-up (1FU) 19.8 ± 3.4 months; 1FU to second follow-up (2FU) 23.6 ± 2.0 months). Persistent MPS was present in 52 participants (11 %). The remaining individuals were defined as controls.

At baseline, individuals with persistent MPS compared with the control cohort were more often male (65 % vs. 43 %; *p* < 0.01), older (67 years vs. 62 years; *p* < 0.01), more often had RBD (35 % vs 19 %; *p* = 0.01), had fewer years of education (13 years vs. 14 years; *p* = 0.03) and less often reported a positive family history for dementia (23 % vs. 39 %; *p* = 0.03; see Table 1). Participants with persistent MPS did not significantly differ from controls in terms of prevalence of hyposmia, occurrence of lifetime depression, severity of depressive symptoms, *ApoE*4 status and frequency of vascular co-morbidities. Table 1 and Additional file 3 present a complete overview of all parameters. According to the grouping criteria, MDS-UPDRS III was significantly higher in individuals with MPS (4 points vs. 0 points; *p* < 0.001).

**Table 1:** Baseline characteristics of participants with persistent MPS and controls

	Controls (*n* = 428)	Persistent MPS (*n* = 52)	*p* value
Male gender (*N* (%))	182 (43)	34 (65)	0.002
Age (years)	62 (50–80)	67 (51–77)	<0.001
	(62 ± 7)	(67 ± 6)	
Education (years)	14 (9–21)	13 (9–21)	0.030
	(15 ± 3)	(14 ± 3)	
Family history PD (%)	63 (15)	8 (15)	0.838
Family history dementia (%)	167 (39)	12 (23)	0.033
MMSE (0–30)	29 (25–30)	29 (25–30)	0.173
	(29 ± 1)	(28 ± 1)	
MDS-UPDRS-III (0–132)	0 (0–10)	4 (2–17)	<0.001
	(1 ± 2)	(5 ± 3)	
Lifetime depression (%)	155 (36)	20 (39)	0.762
Hyposmia (%)	95 (22)	15 (29)	0.216
RBD (%)	81 (19)	18 (35)	0.011
BDI-I (0–63)	6 (0–38)	6 (0–38)	0.121
	(7 ± 6)	(9 ± 7)	
Hyperechogenic substantia nigra (%)	106 (25)	19 (37)	0.075
*ApoE*4 positive (%)	94 (22)	12 (23)	0.931

Data are presented as median (range) (mean ± standard deviation) or number (percentage of total)

*ApoE*4 apolipoprotein 4, *BDI-I* Beck Depression Inventory I, *MMSE* Mini Mental State Examination, *MPS* mild parkinsonian signs, *PD* Parkinson’s disease, *RBD* rapid eye movement sleep behaviour disorder, *MDS-UPDRS-III* Unified Parkinson’s Disease Rating Scale part 3

Individuals with persistent MPS showed worse cognitive performance compared with controls in the CERAD total score, TMT-A and TMT-B at all three time points. Further, they showed a non-significant trend to be worse in ΔTMT-B – TMT-A at baseline. This difference became significant within the follow-up evaluations (Table 2). During the follow-up period (BL to 2FU), controls had stable results in the longitudinally assessed CERAD total scores (*p* = 0.210) and TMT-A (*p* = 0.422) but an improvement in their performance in the TMT-B (*p* < 0.001). In contrast, individuals with persistent MPS showed stable results only in the TMT-A (*p* = 0.684) but tended (non-significant trend) to deteriorate in the TMT-B (*p* = 0.078) and in the CERAD total score (*p* = 0.062) during the follow-up period. The two groups did not differ in the memory, language or visuospatial domain of the CERAD-Plus battery but differed in the executive domain, represented by the TMT.

**Table 2:** Cognitive test performance in controls and individuals with persistent MPS

	Examination	Controls	Persistent MPS	*p* value
CERAD total score (0–100)	BL	87 (64–100)	84 (60–96)	0.002^a^
		(87 ± 7)	(83 ± 8)	
	1FU	86 (56–100)	82 (44–97)	0.002^a^
		(85 ± 8)	(80 ± 8)	
	2FU	88 (57–100)	83 (50–98)	<0.001^a^
		(87 ± 8)	(82 ± 9)	
TMT-A (s)	BL	34 (15–100)	39 (24–81)	0.005^a^
		(36 ± 12)	(41 ± 12)	
	1FU	34 (17–103)	41 (21–71)	0.016^a^
		(36 ± 14)	(43 ± 12)	
	2FU	33 (15–90)	40 (24–110)	0.014^a^
		(36 ± 11)	(43 ± 14)	
TMT-B (s)	BL	80 (34–300)	96 (47–300)	0.050^a^
		(88 ± 33)	(105 ± 40)	
	1FU	76 (26–300)	93 (55–300)	<0.001^a^
		(82 ± 34)	(107 ± 49)	
	2FU	73 (25–300)	97 (47–300)	0.001^a^
		(81 ± 37)	(112 ± 61)	
TMT-B – TMT-A (s)	BL	46 (11–185)	54 (8–206)	0.083^a^
		(52 ± 30)	(64 ± 40)	
	1FU	40 (5–243)	51 (13–229)	0.001^a^
		(46 ± 29)	(64 ± 43)	
	2FU	39 (0–230)	52 (19–265)	0.004^a^
		(46 ± 33)	(70 ± 54)	
Executive domain	BL	0.406 ± 1.0	0.136 ± 1.0	0.288
	1FU	0.690 ± 1.2	0.215 ± 1.1	0.010
	2FU	0.887 ± 1.3	0.368 ± 1.1	0.014
Memory domain	BL	0.002 ± 0.8	0.028 ± 0.8	0.862
	1FU	0.177 ± 0.8	0.002 ± 0.8	0.157
	2FU	0.169 ± 0.9	0.124 ± 0.8	0.463
Language domain	BL	0.416 ± 0.7	0.370 ± 0.7	0.956
	1FU	0.004 ± 0.7	−0.042 ± 0.7	0.575
	2FU	0.343 ± 0.7	0.121 ± 0.7	0.124
Visuospatial domain	BL	0.284 ± 0.9	0.221 ± 1.3	0.073
	1FU	0.431 ± 0.9	0.074 ± 1.3	0.697
	2FU	0.041 ± 1.0	0.488 ± 1.2	0.115

Values are presented as median (range) (mean ± standard deviation) for the CERAD total score and the TMT. For the four subdomains, values are presented as mean ± standard deviation of demographically adjusted *z* scores

^a^ *p* values were corrected for age, gender and years of education

*BL* baseline, *CERAD* Consortium to Establish a Registry for Alzheimer’s Disease, *1FU* first follow-up, *2FU* second follow-up, *MPS* mild parkinsonian signs, *TMT* Trail Making Test

The overall relative risk of individuals with persistent MPS to develop cognitive impairment (defined as being in the fourth quartile with more than >98 sec needed for completion of TMT-B at the 2FU) was 2.06 (95 % confidence interval: 1.45–2.92; *p* < 0.001).

**Fig. 1:**
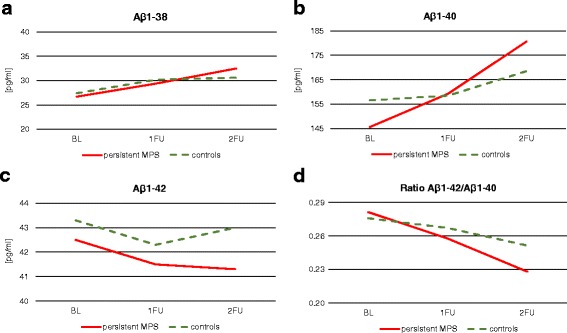
Levels of plasma Aβ_1–38_, Aβ_1–40_ and Aβ_1–42_ in individuals with mild parkinsonian signs (*MPS*) and controls. **a** Aβ_1–38_, **b** Aβ_1–40_, **c** Aβ_1–42_ and **d** ratio Aβ_1–42_/Aβ_1–40_. Individuals with persistent MPS had significantly lower Aβ_1–42_ values than controls at all three time points. In addition, Aβ_1–42_ values and the ratio Aβ_1–42_/Aβ_1–40_ of persistent MPS participants decreased over time, whereas the control group remained stable. No significant differences between controls and individuals with persistent MPS were found for Aβ_1–38_ and Aβ_1–40_ values either cross-sectionally or longitudinally. *Aβ* amyloid-beta, *BL* baseline, *1FU* first follow-up, *2FU* second follow-up

After correction for age and gender, individuals with persistent MPS had significantly lower Aβ_1–42_ values than controls at all time points (BL, *p* = 0.007; 1FU, *p* = 0.022; 2FU, *p* = 0.021). In addition, Aβ_1–42_ values of persistent MPS participants decreased over time, whereas the control group remained stable over the three time points (*p* = 0.169). Similar results were found for the ratio Aβ_1–42_/Aβ_1–40_. Compared with controls in individuals with persistent MPS, the Aβ_1–42_/Aβ_1–40_ ratio tended to be lower at 1FU (*p* = 0.067) and was significantly decreased at 2FU (*p* = 0.033). No significant differences were found between controls and individuals with persistent MPS for Aβ_1–38_ and Aβ_1–40_ values either cross-sectionally or longitudinally (Fig. 1).

Further, we found highly significant correlations between the “diagnosis” of MPS and plasma level of Aβ_1–42_, CERAD total score and TMT (Table 3).

**Table 3:** Correlation between persistent MPS and plasma levels of amyloid-beta and cognitive test performance

	Pearson’s *r* coefficient
Amyloid-beta_1–38_	−0.04
Amyloid-beta_1–40_	−0.02
Amyloid-beta_1–42_	−0.14**
CERAD total score	−0.16***
TMT-A	0.17***
TMT-B	0.16***
TMT-B – TMT-A	0.13***

**p < 0.01,***p < 0.001

*CERAD* Consortium to Establish a Registry for Alzheimer’s Disease, *MPS* mild parkinsonian signs, *TMT* Trail Making Test

## Discussion

The main findings of this prospective longitudinal study are the associations of MPS with both cognitive deficits and low plasma Aβ_1–42_ levels, as well as the progression of cognitive deterioration and plasma Aβ_1–42_ reduction in individuals with MPS but not in those without.

In this prospective study of neurologically healthy older adults, the phenomenon of persistent MPS is associated with higher age and lower education but not with other known risk factors for dementia such as *ApoE*4 positive status, family history for dementia or vascular diseases. This is in line with a large prospective study which found no association of MPS with these risk factors but still found an increased risk for dementia in individuals with baseline MPS [4].

The cognitive profile of participants with persistent MPS was worse than that of the control group. Individuals with persistent MPS were significantly worse in the cognitive tests at all time points investigated (except for ΔTMT-B–TMT-A at baseline) and further showed a non-significant trend for cognitive decrease over time. These results suggest that individuals with persistent MPS might be in a prodromal phase of dementia and might progress to more severe cognitive conditions.

Aβ_1–42_ is probably the most studied biofluid marker for the prediction of cognitive decline or dementia in neurodegenerative research. We found that individuals with persistent MPS had reduced plasma levels of Aβ_1–42_ at all study time points and that their levels decrease during the observational period. Taking into account that cerebrospinal fluid (CSF) Aβ_1–42_ levels are reduced to a level indicative for AD 5–10 years before the onset of dementia [28], with the first changes estimated to occur more than two decades before related cognitive decline [29], one could suggest that the reduced levels of Aβ_1–42_ in the plasma of individuals with persistent MPS are somehow related to CSF changes of Aβ_1–42_. Consequently, a reduction of plasma Aβ_1–42_ may be related to the prodromal phase of AD. In contrast to CSF Aβ_1–42_, there are only few studies on plasma Aβ_1–42_ with inconsistent results. In cross-sectional studies of individuals with AD and mild cognitive impairment, lower [30], unchanged [31, 32] or higher [33] levels of Aβ_1–42_ have been found. A problem of these cross-sectional studies is the relatively low number of participants. The even fewer longitudinal studies measured Aβ in healthy individuals only once at baseline to evaluate the risk of AD, but they did not evaluate the course of Aβ in the prodromal phase. For example, Sundelof et al. found an association of reduced Aβ_1–42_ and increased risk of AD while Mayeux et al. found an association between high levels of Aβ_1–42_ and increased risk for AD [34, 35]. Our study is therefore the first reporting repeated measurements of Aβ_1–42_.

Although plasma Aβ_1–42_ changes are small, the finding may still be of relevance because probably a (small) subgroup of individuals with persistent MPS will develop AD. It is also possible that the reduction of Aβ_1–42_ plasma levels in blood occur later than in CSF. Further follow-up of this study and other studies measuring plasma levels of Aβ_1–42_ would be of great interest because collection of blood is easier, less invasive and can be done in greater cohorts than collection of CSF.

The strength of this study is the prospective longitudinal study design. Moreover, our MPS cohort only included individuals with persistent MPS over the whole observation period, thereby reducing confounders such as individual daily condition (e.g. well rested, mood or nervousness) and inter-rater differences. Most former studies defined their cohort based on one single evaluation but motor signs, especially when they are less distinct, can fluctuate. By taking only individuals with persistent MPS into account, we reduced the amount of false positives and analysed a more specific group at risk for neurodegeneration.

A limitation of this analysis is the relatively short follow-up period of a median of 3.7 years. However, we were able to find significant differences in cognition and Aβ_1–42_ levels. It is highly probable that these effects become even stronger with a longer follow-up period. Moreover, despite the possible influence of depression on cognitive performance, we did not exclude participants which might be clinically depressed (BDI >18), because the number of those severely depressed individuals was low and equally distributed between the two groups (MPS 9 % and HC 7 %). Therefore, we do not think that the results will be influenced substantially. Additional studies are warranted for further validation.

## Conclusion

Taken together, low cognitive performance and plasma Aβ_1–42_ levels which decrease over time seem to be associated with persistent MPS. This suggests that, at least in a statistically relevant subgroup of individuals with persistent MPS, an underlying neurodegenerative process associated with AD pathology may be present. The potential of the “syndrome” MPS as a predictive marker for future neurodegenerative diseases should therefore be further explored.

## Additional files

Additional file 1: is Table S1 presenting test specifications and characteristics for the methodology. (DOCX 17 kb)
Additional file 2: is a figure showing ELISA test run monitoring. Three QC samples were included (single testing) in parallel with the test samples. Only runs with available data points for the three samples were considered. The coloured blocks show the 95 % confidence interval (±2 SD). (PPTX 8306 kb)
Additional file 3: is Table S2 presenting surrogate markers of risks and co-morbidities. Participants with persistent MPS did not significantly differ from controls in terms of frequency of vascular co-morbidities. (DOCX 11 kb)

## Abbreviations

1FU: First follow-up
2FU: Second follow-up
AD: Alzheimer’s disease
ApoE4: Apolipoprotein 4
Aβ: Amyloid-beta
BDI-I: Beck Depression Inventory I
BL: Baseline
CCA: Common carotid artery
CERAD: Consortium to Establish a Registry for Alzheimer’s Disease
CSF: Cerebrospinal fluid
IMT: Intima-media thickness
MMSE: Mini Mental State Examination
MPS: Mild parkinsonian signs
PD: Parkinson’s disease
QC: Quality control
RBD: Rapid eye movement sleep behaviour disorder
RBDSQ: RBD screening questionnaire
SD: Standard deviation
TMT: Trail-Making Test
TREND: Tübinger Evaluation of Risk Factors for Early Detection of Neurodegenerative Disorders
UPDRS: Unified Parkinson’s Disease Rating Scale
