# Supplementary material for: Prospective longitudinal course of cognition in older subjects with mild parkinsonian signs
Source: Alzheimers Res Ther. 2016 Oct 10;8:42. doi: 10.1186/s13195-016-0209-7 (PMC5057460; doi:10.1186/s13195-016-0209-7)
Supplement: Additional file 1: — is Table S1 presenting test specifications and characteristics for the methodology. (DOCX 17 kb) [file 13195_2016_209_MOESM1_ESM.docx]

Supplemental table 1: Test specifications and characteristics for methodology

|  |  | | |  | **Euroimmun β-Amyloid plasma ELISAs** | | | | |
| --- | --- | --- | --- | --- | --- | --- | --- | --- | --- |
| **ANALYTE** | | | | | Aβ1-38 | | Aβ1-40 | | Aβ1-42 |
| REGULATORY STATUS | | | | | RUO | | | | |
| MATRIX | | | | | EDTA plasma | | | | |
| TECHNOLOGY | | | | | ELISA 96 well ABSORBANCE PLATE READER (450-630 nm) | | | | |
| BIOMATERIALS | Capture mAb | Name | | | ADx104 (4H9) | ADx103 (2G3) | | ADx102 (21F12) | |
|  |  | Isotype | | | IgG1 | IgG1 | | IgG2a | |
|  |  | Epitope | | | Aβ x-38 | Aβ x-40 | | Aβ x-42 | |
|  | Detector mAb | Name | | | ADx101 (3D6) | | | | |
|  |  | Isotype | | | IgG2b | | | | |
|  |  | Epitope | | | Aβ 1-x | | | | |
|  | Calibrator | Type | | | Recombinant protein | | | | |
|  |  | Source | | | rPeptide | | | | |
| ASSAY | Sample incubation | Simultaneouos/ sequentialwith detector mAb | | | Simultaneous | | | | |
|  |  | Volume Sample (µL) | | | 80 | | | | |
|  |  | Volume Detector (µL) | | | 20 | | | | |
|  |  | Total volume/well | | | 100 | | | | |
|  |  | Pre-dilution | | | Yes (1/4) | | | | |
|  |  | % Plasma/well | | | 20 | | | | |
|  |  | Recipient for pre-dilution | | | PP (96 well microtiter-plate) | | | | |
|  | Boundary Conditions | Sample Incubation | | | 3 hours | | | | |
|  |  |  |  |  | RT | | | | |
|  |  | Detection incubation | | | 0.5 hours | | | | |
|  |  |  |  |  | RT | | | | |
|  |  | Substrate incubation | | | 0.5 hours | | | | |
|  |  |  |  |  | RT | | | | |
|  | Calibrators | Format | | | Ready-to-use | | | | |
|  |  | Design | | | Lyophilized Stable at RT | | | | |
|  |  | Number | | | 7 non-zero + BLANK | | | | |
|  |  | Concentration range (pg/mL) *(lot specific)* | | | 0.5-45 | 7.5-75 | | 1-40 | |
|  |  | Curve-fit algorithm | | | 4PL/5PL | | | | |
| AUTOMATION | | | | | Validated | | | | |
| ASSAY PERFORMANCE (analytical) | Sensitivity  (CLSI EP17-A) | | **Limit of Blank** LoB (pg/mL) | | 0.4  (Range: 0.3-0.5 ) (n=24 wells; 4 Runs) | 1.4  (Range: 1.0-1.8) (n=24 wells; 4 Runs) | | 1.2  (Range: 1.0-1.4) (n=24 wells; 4 Runs) | |
|  |  |  | **Limit of Detection** LOD (pg/mL) | | 1.6 (Range: 1.5-1.7) (n=24 wells; 4 Runs) | 5.7 (Range: 5.3-6.1) (n=24 wells; 4 Runs) | | 2.4  (Range: 2.1-2.6) (n=24 wells; 4 Runs) | |
|  |  |  | **Limit of Quantitation** LOQ (pg/mL) | | 2.3 (n=12 wells; at 1.3-16 pg/ml) | 12.5 (n=12 wells; at 4-36 pg/ml) | | 3.5 (n=12 wells; at 4-12 pg/ml) | |
|  | Precision   (CLSI EP05-A2) | | **Intra-assay** (% CV) | | 6.9 % (range: 2.2-6.9 %) (n=24 wells, at 16-213 pg/ml) | 3.5 % (range: 1.8-6.6 %) (n=24 wells, at 31-231 pg/ml) | | 4.3 % (range: 2.2-6.9 %) (n=24 wells, at 13-124 pg/ml) | |
|  |  |  | **Inter-assay** (%CV) | | 15.5 % (8.1-23.1 %) (10 runs; duplicates, 11-160 pg/ml) | 5.3 % (3.1-6.5 %) (10 runs; duplicates, 32-238 pg/ml) | | 11.2 % (7.4.-19.5 %) (10 runs; duplicates, 6-124 pg/ml) | |
|  | Parallelism   (CLSI EP6-A) | |  | | Parallelism is demonstrated | Parallelism is demonstrated | | Parallelism is demonstrated | |
|  | Specificity | | Testing of Aß isoforms | | Aß1-38 Aß1-43 (<4.1%) | Aß1-40 Aß1-39 (<0.8%) | | Aß1-42 Aß1-43 (<4.1%) | |

Abbreviations: RT, Room temperature; RV, Run-validation (= calibrator in buffer); PP, Polypropylene; LOB, Limit Of Blank; LOD, Limit Of Detection; LOQ, Limit Of Quantification
