# Supplementary figures and images for: Prospective longitudinal course of cognition in older subjects with mild parkinsonian signs
Source: Alzheimers Res Ther. 2016 Oct 10;8:42. doi: 10.1186/s13195-016-0209-7 (PMC5057460; doi:10.1186/s13195-016-0209-7)

## Slide 1
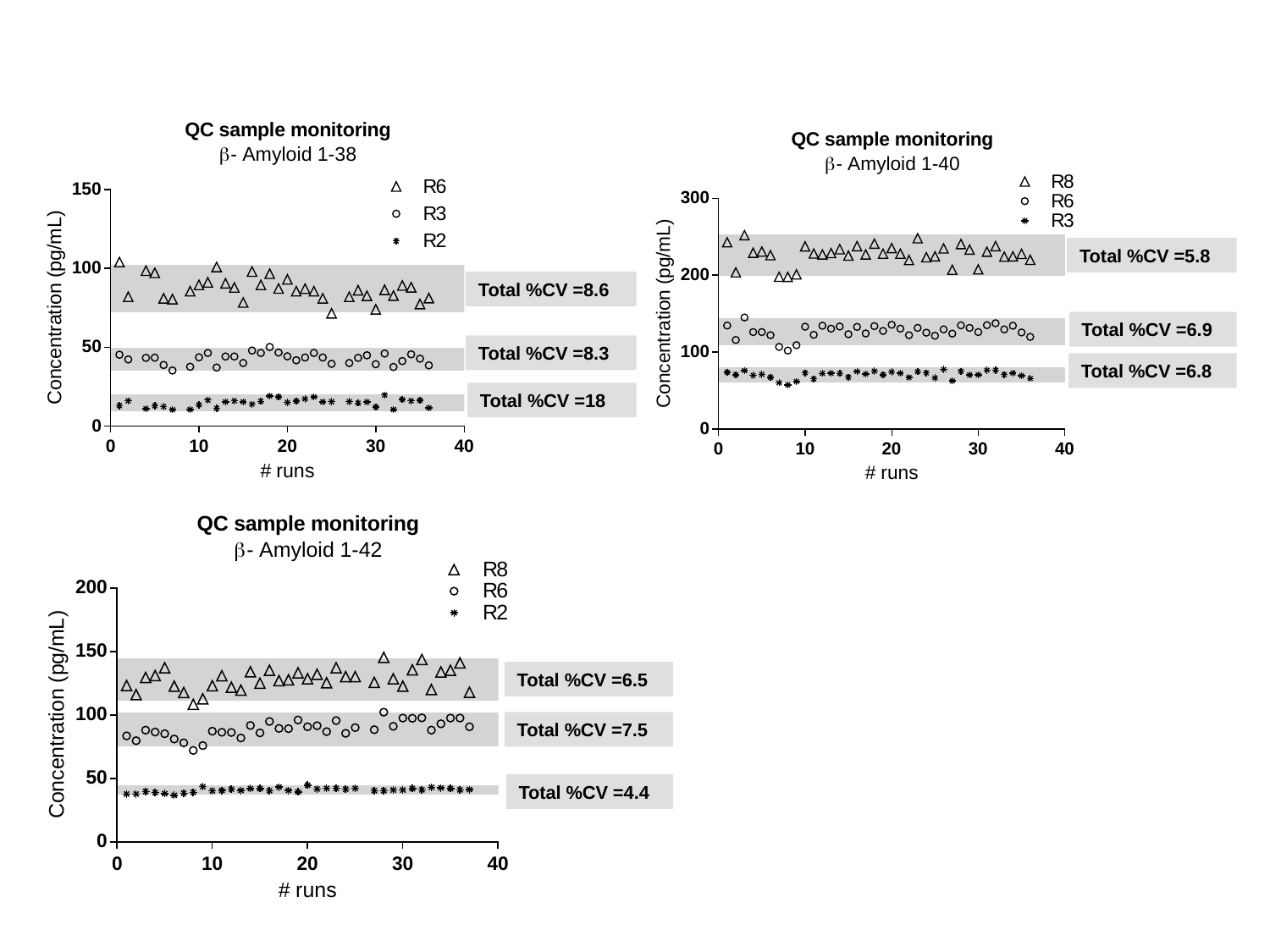

Total %CV =8.6
Total %CV =8.3
Total %CV =18
Total %CV =5.8
Total %CV =6.9
Total %CV =6.8
Total %CV =6.5
Total %CV =7.5
Total %CV =4.4

Supplement: Additional file 2: — is a figure showing ELISA test run monitoring. Three QC samples were included (single testing) in parallel with the test samples. Only runs with available data points for the three samples were considered. The coloured blocks show the 95 % confidence interval (±2 SD). (PPTX 8306 kb) [file 13195_2016_209_MOESM2_ESM.pptx]
