# Supplementary material for: Prospective longitudinal course of cognition in older subjects with mild parkinsonian signs
Source: Alzheimers Res Ther. 2016 Oct 10;8:42. doi: 10.1186/s13195-016-0209-7 (PMC5057460; doi:10.1186/s13195-016-0209-7)
Supplement: Additional file 3: — is Table S2 presenting surrogate markers of risks and co-morbidities. Participants with persistent MPS did not significantly differ from controls in terms of frequency of vascular co-morbidities. (DOCX 11 kb) [file 13195_2016_209_MOESM3_ESM.docx]

Table S2: Surrogate markers of risks and comorbidities

|  | **Controls**  **n=428** | **persistent MPS n=52** | **p-value** |
| --- | --- | --- | --- |
| **Intima Media Thickness [mm]** | 0.75 (0.4-1.3) | 0.80 (0.5-1.2) | 0.081 |
| **Hypertension [%]** | 139 (32) | 20 (38) | 0.436 |
| **Heart Disease [%]** | 93 (22) | 8 (15) | 0.368 |
| **Stroke or TIA [%]** | 2 (1) | 1 (2) | 0.292 |
| **Diabetes mellitus [%]** | 31 (7) | 1 (2) | 0.235 |

Data are presented with median (range) or number (percentage of total). Heart disease included: heart rhythm disturbance, heart attack, congestive heart failure and coronary heart disease.

MPS, Mild Parkinsonian Signs, TIA, transient ischemic attack
